# Associations between CT-determined visceral fat burden, hepatic steatosis, circulating white blood cell counts and neutrophil-to-lymphocyte ratio

**DOI:** 10.1371/journal.pone.0207284

**Published:** 2018-11-20

**Authors:** Kuo-Tzu Sung, Richard Kuo, Jing-Yi Sun, Ta-Chuan Hung, Shun-Chuan Chang, Chuan-Chuan Liu, Chun-Ho Yun, Tung-Hsin Wu, Chung-Lieh Hung, Hung-I Yeh, Charles Jia-Yin Hou, Ricardo C. Cury, David A. Zidar, Hiram G. Bezerra, Chris T. Longenecker

**Affiliations:** 1 Department of Medicine, Mackay Medical College, Taipei, Taiwan; 2 Mackay Junior College of Medicine, Nursing and Management, Taipei, Taiwan; 3 Division of Cardiology, Department of Internal Medicine, Mackay Memorial Hospital, Taipei, Taiwan; 4 Department of Radiology, Mackay Memorial Hospital, Taipei, Taiwan; 5 Department of Biomedical Imaging and Radiological Sciences, National Yang Ming University, Taipei, Taiwan; 6 Department of Medical Technology, Yuanpei University of Science and Technology, Hsin-Chu, Taiwan; 7 Graduate Institute of Health Care Organization Administration, College of Public Health National Taiwan University, Taipei, Taiwan; 8 Health Evaluation Center, Mackay Memorial Hospital, Taipei, Taiwan; 9 Cardiovascular MRI and CT Program, Baptist Cardiac Vascular Institute, Miami, Florida, United States of America; 10 Division of Cardiology, Department of Internal Medicine, University Hospitals Harrington Heart & Vascular Institute, Case Western Reserve University, Cleveland, OH, United States of America; Weill Cornell Medical College in Qatar, QATAR

## Abstract

Visceral adiposity is associated with cardiovascular disease, an association that may be mediated in part by inflammation. We hypothesized that regional measures of visceral adiposity would associate with commonly obtained clinical measures of immune status. We consecutively studied 3,291 subjects (mean age, 49.8±9.8 years) who underwent an annual cardiovascular risk survey. Peri-cardial (PCF) and thoracic peri-aortic adipose tissue (TAT) volumes were determined by dedicated computed tomography (CT) software (Aquarius 3D Workstation, TeraRecon, San Mateo, CA, USA). Hepatic steatosis was assessed by abdominal ultrasonography. We explored cross-sectional associations between visceral fat measures and high-sensitivity C-reactive protein (hs-CRP), leukocyte counts, and the neutrophil-to-lymphocyte ration (NLR). Among 3,291 study participants, we observed positive linear associations between PCF and TAT, higher degree of hepatic steatosis and hs-CRP, various leukocyte counts, either total and its differential counts, and NLR (all trend p<0.001). Multi-variate linear and logistic regression models showed independent associations between PCF/TAT (ß-Coef: 0.14/0.16, both p<0.05) and total WBC counts, with only TAT further demonstrated significant relations with neutrophil counts and NLR (both p<0.05) and independently identified abnormally high WBC and NLR (Odds ratio: 1.18 & 1.21, both p<0.05). C-statistics showed significant incremental model prediction for abnormally high WBC and NLR (both ΔAUROC<0.05) when TAT was superimposed on traditional cardiovascular risks and biochemical information. Greater visceral adiposity burden and hepatic steatosis may be associated with higher circulating leukocyte counts and markers for atherosclerosis, with more pronounced influences for peri-aortic adiposity. Our data suggested the differential biological impacts for region-specific visceral adiposity.

## Introduction

Excessive adipose tissue is associated with a chronic inflammatory status that leads to a variety of metabolic disorders including dyslipidemia, hypertension, and type 2 diabetes with region-specific properties [1]. Compared to subcutaneous adipose tissue, visceral adipose tissue is functionally more active in several metabolic derangements and may not be accurately identified by traditional anthropometrics including body weight and body mass index (BMI) assessment [2]. Over-production of various pro-inflammatory paracrines and mediators by visceral adipose tissue leads to obesity-related systemic inflammation and insulin resistance [3, 4]. On the other hand, fatty liver disease—characterized by excessive fatty infiltration of liver tissue—is an increasingly recognized cause of chronic liver disease worldwide, and is highly associated with central obesity, local hepatic inflammation, insulin resistance and increased systemic oxidative stress [5, 6].

Typically, the local inflammatory milieu of visceral adipose tissue is characterized by monocyte/macrophage infiltration and a diversity of lymphocyte subtypes [7, 8]. Additionally, increasing evidence suggests that various adipokines, free radicals from exaggerated oxidative stress and proinflammatory cytokines secreted directly from adipocytes may have remote adverse cardiovascular effects in obesity [9]. Although cytokines are rarely measured in daily practice, the total white blood cell count (WBC) and its subtypes (e.g. monocytes, lymphocytes, neutrophils, eosinophils, and basophils) may reflect a patient`s inflammatory status in the absence of infection [10]. In particular, the neutrophil-to-lymphocyte ratio (NLR) has been proposed as a risk marker for adverse inflammatory status in metabolic syndrome, several cardiovascular disorders and cancer [11].

Recently, two volume-based measures of visceral adiposity burden—peri-cardial (PCF) and thoracic peri-aortic adipose tissue (TAT)—have been shown to correlate with metabolic risk profiles and atherosclerosis [12]. Studies over the past decade also suggest that both PCF and TAT are independently associated with systemic inflammatory markers such as hs-CRP. As region-specific visceral adiposity may have biologically diverse effects and associate with different pathological conditions [13, 14], the aim of this study was to investigate to what extent these visceral fat measures (including PCF, TAT and hepatic steatosis) may be associated with circulating leukocyte counts and the NLR in a large cohort of adults in Taiwan.

## Methods

### Study population

From 2005 to 2009, we studied consecutive subjects who underwent a comprehensive cardiovascular health survey at our center that included a non-contrast computed tomography (CT) scan of the chest to assess the presence and burden of coronary calcium. Subjects with CT data available comprised the main study population in our current work. This study complies with the Declaration of Helsinki, and the study protocol was approved by the Institutional Review Board at Mackay Memorial Hospital, Taipei, Taiwan. Data were analyzed anonymously.

Baseline demographics and medical history were obtained along with a detailed physical exam. Structured questionnaires were used to quantify self-reported alcohol consumption, smoking and physical activity. Presence of coronary arterial disease (CAD) was defined as a history of myocardial infarction or prior history of coronary revascularization. History of hypertension was defined as either systolic blood pressure higher than 140mmHg, diastolic pressure higher than 90mmHg or previous diagnosed hypertension on treatment. Diabetes was defined as current usage of any medications for diabetes or known diagnosis of diabetes. Hyperlipidemia was defined as known history of hyperlipidemia or medications (e.g. statin or fibrate). Anthropometric measures including height, weight, waist and hip circumferences were all obtained. Resting blood pressures were measured by medical staff using a standardized automated sphygmomanometer.

### Biochemical data, white blood cell counts and serum markers of systemic inflammation

Venous blood sampling and analysis were performed according to the Clinical Laboratory Standards Institute guidelines (Specimen Choice, Collection, and Handling; Approved Guideline H18-A3). Levels of total white blood cell (WBC) counts (leukocytes), neutrophils, lymphocytes, and monocytes were all determined by an automated blood cell counter utilizing Coulter LH780 Hematology Analyzer (Beckman Coulter Ireland Inc Mervue, Galway, Ireland). In our current work, absolute cell counts were used in the analyses. To ensure accuracy, results were verified by repeating the tests on the same tube one day later. A Hitachi 7170 Automatic Analyzer (Hitachi Corp. Hitachinaka Ibaraki, Japan) was used to measure fasting glucose, post-prandial glucose, HbA1c, uric acid, blood urea nitrogen, creatinine, homocysteine, and several lipid profiles including HDL, LDL, total cholesterol and triglyceride, with estimated glomerular filtration rate (eGFR) calculated using the Modification of Diet in Renal Disease equation. High-sensitivity CRP (hs-CRP) levels were determined using a highly sensitive, latex particle-enhanced immunoassay Elecsys 2010 (Roche, Mannheim, Germany).

### Measurements of ectopic visceral adipose tissue

Scans were performed using a 16-slice multidetector CT (MDCT) scanner (Sensation 16; Siemens Medical Solutions, Forchheim, Germany) with 16 × 0.75 mm collimation, rotation time of 420 msec, and tube voltage of 120 kV. In one breath hold, images were acquired from above the level of tracheal bifurcation to below the base of heart using prospective electrocardiographic triggering, with the center of the acquisition at 70% of the R-R interval. From the raw data, the images were reconstructed with standard kernel in 3 mm thick axial, non-overlapping slices and 25 cm field of view.

**Fig 1:**
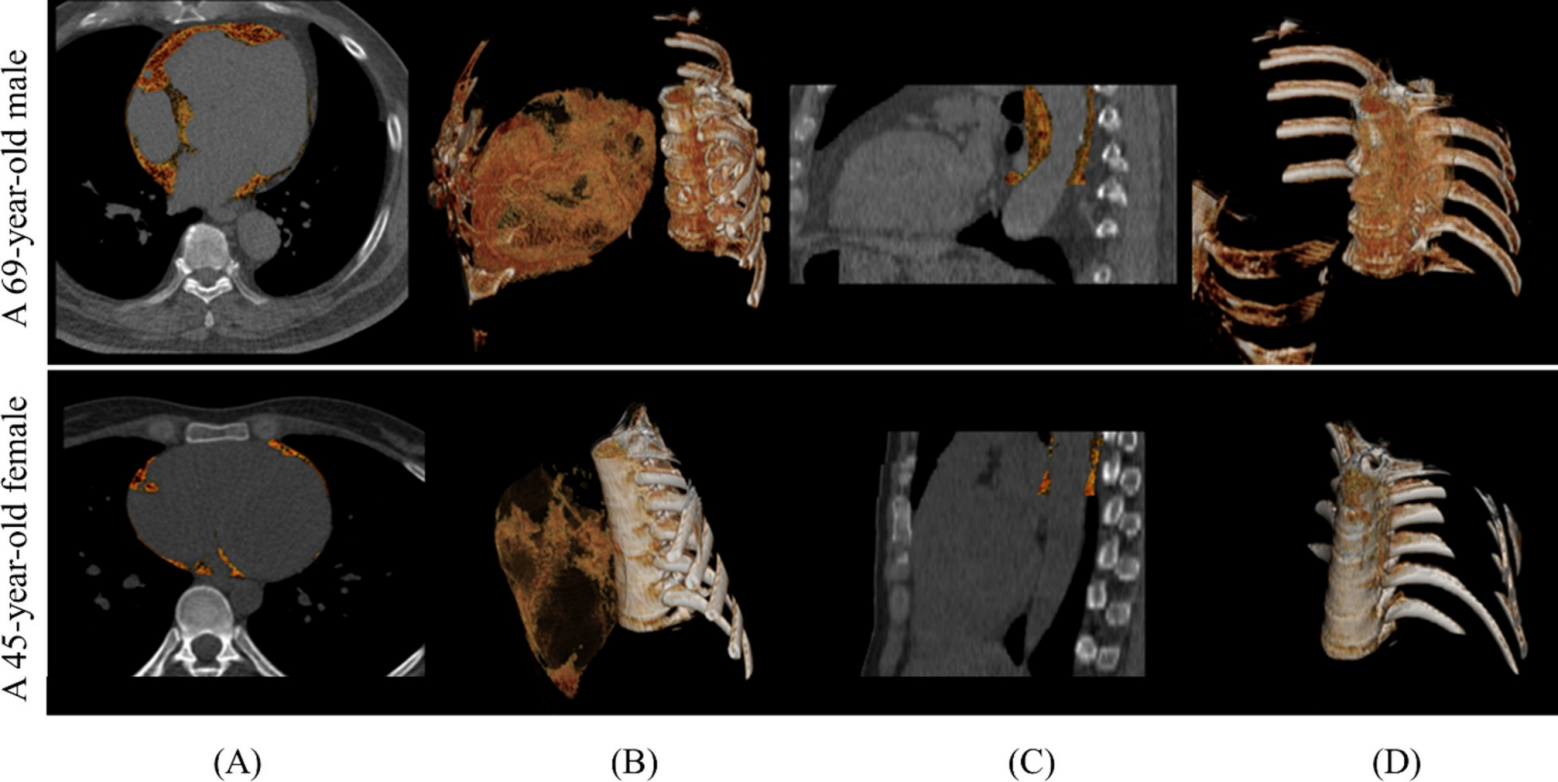
**Examples of PCF (A, B) and TAT (C, D) in 2D and 3D computed tomography views.** A 69-year-old male with large amounts of PCF (235.8ml), TAT (24.9ml) and the lab data (WBC: 10700/mm^3^, Neutrophil: 9502/mm^3^, NLR: 15.05, monocyte: 481/mm^3^) (First row). A 45-year-old female with small amounts of PCF (18.7ml), TAT (1.24ml) and the lab data (WBC: 3900/mm^3^, Neutrophil: 2211/mm^3^, NLR: 1.702, monocyte: 238/mm^3^) (Second row). PCF: peri-cardial adipose tissue; TAT: thoracic peri-aortic adipose tissue; WBC: white blood cell count; NLR: neutrophil-to-lymphocyte ratio.

PCF volumes were quantified from the heart CT scan using a dedicated workstation (Aquarius 3D Workstation, TeraRecon, San Mateo, CA, USA). The semi-automatic segmentation technique was developed for quantification of adipose tissue volumes. We traced pericardium in axial MDCT images manually from the level of left main coronary artery to diaphragm every four to six slices. The computer software then automatically interpolated and traced pericardium along the manually traced areas. All automatically traced slices were verified and modified if necessary for accuracy. Adipose tissue was defined as pixels within a window of -195 to -45 HU and a window center of -120 HU. Pericardial fat (PCF) was defined as any adipose tissue located within the pericardial sac. Thoracic peri-aortic adipose tissue (TAT) was defined as all of the adipose tissue surrounding the thoracic aorta extending 67.5 mm caudally from the level of the bifurcation of pulmonary arteries. (Fig 1) This approach has previously been validated [15, 16]. Two observers performed independent readings on a random subset of 40 subjects. The intra-observer and inter-observer coefficient of variation were 4.27%, 4.87% and 6.58%, 6.81% for PCF and TAT, respectively [15].

### Ultrasonographic assessment on grade of hepatic steatosis

Hepatic ultrasonography was performed in all patients by experienced gastroenterologists, who were blinded to the patients’ clinical data. Hepatic steatosis was diagnosed based on characteristic ultrasonographic characteristics, including diffuse hyper echogenicity of the liver relative to the kidneys, ultrasonography beam attenuation, and poor visualization of the intrahepatic vessel borders and diaphragm [17]. Ultrasonography allows detection of the presence of mild and moderate-to-severe hepatic steatosis as alternative surrogate of intra-abdominal adiposity, with a sensitivity and specificity of approximately 85% and 95%, respectively (when liver fat infiltration on histology is at least 20–30%)[17]. We categorized hepatic steatosis severity (none, mild, moderate, severe) based on the intensity of hepatic hyper echogenicity compared to the kidney.

### Statistical analysis

Baseline demographics were compared across quintiles of PCF and TAT volumes, and the Wilcoxon rank-sum test was used to estimate the statistical significance of trends across all ordered groups. The prevalence of hepatic steatosis according to PCF/TAT quintiles was visualized using bar graphs, and compared across categories using (chi-squared tests). The associations between leukocyte counts and PCF/TAT were explored using Pearson’s correlation, and logistic regression was used to examine the association of leukocyte counts with moderate or severe hepatic steatosis. We further conducted uni- and multi-variate linear regression models to explore the independent relationships of ectopic fat measures (PCF/TAT and hepatic steatosis as independent variables) with leukocyte counts (as dependent variables) after accounting for several key baseline clinical co-variates. Receiver operating characteristic curves with c-statistics were used to test the diagnostic performance of PCF/TAT and hepatic steatosis added to age, gender, BMI, biochemical profiles, lifestyle factors, and medical history to identify abnormally high total WBC counts (>6.9 × 10^3^/μL)[18], and NLR (>2.51)[19]. The potential confounding factors for multi-variate (MV) regression models included age, gender, BMI, blood pressure, fasting glucose, lipid profiles, renal function, medical histories of hypertension, diabetes, hyperlipidemia, coronary disease, smoking, and alcohol use.

All data were analyzed using STATA 12.0 (STATA Corp., College Station, Texas). All statistical tests were two-sided with p<0.05 considered to be statistically significant.

## Results

### Baseline characteristics and circulating individual white blood cell counts of study participants

A total of 3,291 subjects met our entry criteria and were enrolled in this study. The mean age was 49.8 ± 9.8 years, with averaged BMI estimated to be 24.7 ± 3.5kg/m^2^ in our current cohort. Baseline demographic data across quintiles of PCF and TAT are displayed in Table 1, with mean (± standard deviation) PCF and TAT volumes of 75.7 (±30.9) and 7.1 (±4.0) ml, respectively. Among 3,196 subjects with liver coding data available, 2,736 (85.6%) had no or mild hepatic steatosis, 277 (8.7%) had moderate and 183 (5.7%) had severe hepatic steatosis.

**Table 1:** Baseline characteristics of the study population by PCF and TAT quintiles.

	PCF Quintiles (ml)						TAT Quintiles (ml)					
	Q1 (<50.1)	Q2 (50.1, 64.4)	Q3 (64.4, 77.6)	Q4 (77.6, 96.5)	Q5 (≥96.5)	p (trend)	Q1 (<3.8)	Q2 (3.8, 5.5)	Q3 (5.5, 7.4)	Q4 (7.4, 9.8)	Q5 (≥9.8)	p (trend)
**Baseline Characters**												
Age, year	45.14±9.28	48.72±8.88	49.88±9.13	51.04±9.50	54±9.95	<0.001	45.99±9.23	48.25±9.4	49.53±9.51	50.82±9.28	54.22±9.58	<0.001
Gender (male), (%)	350(53.19%)	453(68.84%)	502(76.18%)	528(80.24%)	548(83.28%)	<0.001	193(29.24%)	420(63.83%)	539(82.04%)	600(90.91%)	629(95.88%)	<0.001
Body height, cm	163.74±8.28	165.97±8.3	166.90±7.64	166.94±7.78	167.69±7.97	<0.001	161.64±8.02	165.68±8.58	167.17±8.03	168.34±6.65	168.4±7.16	<0.001
Body weight, kgw	59.48±10.09	65.15±9.97	68.73±10.53	71.58±11.51	76.33±12.13	<0.001	56.65±8.68	65.18±9.94	68.80±9.45	72.86±10.32	77.71±11.37	<0.001
BMI, kg/m^2^	22.08±2.71	23.59±2.73	24.59±2.89	25.59±3.15	27.07±3.57	<0.001	21.63±2.55	23.68±2.76	24.59±2.72	25.65±2.96	27.35±3.4	<0.001
SBP, mmHg	117.11±16.28	120.89±16.05	123.99±17.28	123.59±15.83	128.70±16.68	<0.001	115.00±16.04	120.19±16.34	122.94±15.26	125.48±15.49	130.68±17	<0.001
DBP, mmHg	71.49±10.40	74.88±10.79	77.02±10.9	77.21±10.18	79.63±10.21	<0.001	70.34±10.27	73.88±9.98	76.64±9.85	78.33±10.11	81.06±10.78	<0.001
Pulse rate, 1/min	72.17±10.20	71.76±10.82	73.36±9.63	73.1±11.01	73.95±12.03	0.002	72.53±10.21	71.55±11.18	72.96±10.01	73.09±10.73	74.18±11.6	<0.001
**Lab Data**												
Hb, g/dL	13.96±1.60	14.46±1.47	14.65±1.41	14.76±1.31	14.8±1.29	<0.001	13.45±1.51	14.33±1.53	14.73±1.23	15.02±1.15	15.1±1.13	<0.001
Fating glucose, mg/dl	94.18±14.34	98.66±15.87	102.53±23.61	103.89±21.96	108.35±28.4	<0.001	92.88±9.87	98.33±17.47	101.38±21.61	102.72±19.9	112.45±30.91	<0.001
Total cholesterol, mg/dl	193.95±33.13	201.68±39.67	205.12±34.99	204.12±35.97	204.97±37.43	<0.001	196.24±35.23	201.44±36.03	205.19±36.41	204.41±38.8	202.51±35.36	<0.001
Triglyceride, mg/dl	103.7±64.04	129.83±168.89	143.64±88.22	148.42±89.52	169.44±114.91	<0.001	92.4±53.57	122.07±66.86	141.53±87.28	160.51±172.51	178.97±121.24	<0.001
LDL, cholesterol, mg/dl	120.75±29.56	130.10±32.03	134.13±31.61	134.06±32.76	134.32±33.25	<0.001	121.26±31.31	129.80±32.74	135.84±32.36	134.92±31.2	131.80±31.66	<0.001
HDL, cholesterol, mg/dl	59.80±15.81	54.68±14.27	51.92±13.03	50.17±11.97	47.28±11.84	<0.001	63.28±15.83	55.48±13.87	51.30±12.11	47.68±10.35	45.90±10.12	<0.001
sGPT, mg/dL	24.94±29.07	27.18±19.31	31.61±24.4	32.65±23.28	36.63±29.96	<0.001	22.01±25.94	27.36±23.16	30.85±19.57	35.83±31.99	36.98±23.55	<0.001
eGFR, ml/min/1.73m^2^	89.22±14.43	86.63±15.53	85.63±14.65	84.95±14.94	83.76±14.99	0.009	90.10±15.07	87.17±14.44	86.00±14.93	83.89±14.06	82.26±15.41	<0.001
**Medical History**												
History of hypertension, %	45(6.84%)	82(12.46%)	116(17.60%)	115(17.48%)	176(26.75%)	<0.001	38(5.76%)	70(10.64%)	90(13.7%)	117(17.73%)	219(33.38%)	<0.001
History of diabetes, %	14(2.13%)	34(5.17%)	35(5.31%)	35(5.32%)	53(8.05%)	<0.001	11(1.67%)	23(3.5%)	32(4.87%)	39(5.91%)	66(10.06%)	<0.001
History of hyperlipidemia treatment, %	13(1.98%)	30(4.56%)	27(4.1%)	38(5.78%)	56(8.51%)	<0.001	12(1.82%)	23(3.5%)	44(6.7%)	34(5.15%)	51(7.77%)	<0.001
History of CAD, %	12(1.82%)	19(2.89%)	23(3.49%)	36(5.47%)	52(7.9%)	<0.001	11(1.67%)	28(4.26%)	29(4.41%)	26(3.94%)	48(7.32%)	<0.001
Alcohol use, %	29(4.41%)	29(4.41%)	37(5.61%)	45(6.84%)	46(6.99%)	0.1	22(3.33%)	25(3.8%)	36(5.48%)	44(6.67%)	59(8.99%)	<0.001
Current smoker, %	50(7.6%)	55(8.36%)	72(10.93%)	89(13.53%)	96(14.59%)	<0.001	30(4.55%)	53(8.05%)	76(11.57%)	92(13.94%)	111(16.92%)	<0.001

BMI: body mass index; DBP: diastolic blood pressure; eGFR: estimated glomerular filtration rate; Hb: hemoglobin; HDL: high-density lipoprotein; LDL: low-density lipoprotein; SBP: systolic blood pressure; sGPT: serum glutamate-pyruvate transaminase.

Higher quintile of PCF and TAT volume was associated with more advanced age, higher systolic/diastolic blood pressures, increased body height and weight, greater BMI, higher fasting glucose, higher HbA1c, and unfavorable lipid profiles including higher total cholesterol, higher low-density lipoprotein (LDL), lower level of high-density lipoprotein (HDL), and lower estimated glomerular filtration rate (eGFR). Subjects with higher visceral fat depots were more likely to have prevalent hypertension, diabetes, hyperlipidemia and cardiovascular disease (all p<0.05).

### The association between visceral adipose tissue volume, hepatic steatosis and circulating differential white blood cell counts

Higher hs-CRP, total WBC and higher proportions of neutrophils, eosinophils, monocytes, lymphocytes, and myelocytes were observed with increasing amounts of PCF and TAT and with higher grades of liver steatosis (Table 2, all p<0.01).

**Table 2:** The correlation between regional-specific adipose tissue, WBC, various individual white blood cells and NLR by PCF, TAT quintiles and hepatic steatosis.

**A**												
** **	**PCF Quintiles (ml)**						**TAT Quintiles (ml)**					
**Factors**	**Q1** **(<50.1)**	**Q2** **(50.1, 64.4)**	**Q3** **(64.4, 77.6)**	**Q4** **(77.6, 96.5)**	**Q5** **(>96.5)**	**p** **(trend)**	**Q1** **(<3.8)**	**Q2** **(3.8, 5.5)**	**Q3** **(5.5, 7.4)**	**Q4** **(7.4, 9.8)**	**Q5** **(>9.8)**	**p** **(trend)**
hs-CRP, mg/L	0.15±0.19	0.19±0.20	0.19±0.21	0.25±0.27	0.27±0.24	<0.001	0.15±0.19	0.17±0.18	0.21±0.22	0.25±0.25	0.27±0.27	<0.001
WBC count, (10^3^/mm^3^)	5.72±1.39	5.98±1.34	6.07±1.33	6.25±1.38	6.37±1.34	<0.001	5.52±1.37	5.9±1.3	6.09±1.31	6.38±1.34	6.51±1.31	<0.001
Neutrophil count (10^3^/mm^3^)	3.3±1.12	3.43±1.03	3.46±1.01	3.58±1.03	3.64±1.01	<0.001	3.19±1.11	3.33±1.01	3.47±0.98	3.68±1.03	3.73±1	<0.001
Eosinophil count (10^3^/mm^3^)	0.16±0.18	0.15±0.13	0.15±0.14	0.16±0.14	0.18±0.16	<0.001	0.14±0.18	0.15±0.14	0.16±0.13	0.17±0.14	0.18±0.13	<0.001
Basophil count (10^3^/mm^3^)	0.02±0.02	0.02±0.02	0.02±0.02	0.03±0.03	0.02±0.03	0.258	0.02±0.02	0.02±0.02	0.02±0.02	0.02±0.03	0.02±0.03	0.109
Monocyte count (10^3^/mm^3^)	0.39±0.14	0.41±0.14	0.42±0.14	0.42±0.15	0.43±0.15	<0.001	0.37±0.14	0.4±0.14	0.41±0.15	0.43±0.15	0.45±0.14	<0.001
Lymphocyte count (10^3^/mm^3^)	1.83±0.48	1.94±0.51	1.98±0.52	2.03±0.57	2.04±0.55	<0.001	1.78±0.48	1.94±0.49	1.99±0.54	2.04±0.53	2.08±0.56	<0.001
NLR	1.89±0.79	1.86±0.70	1.85±0.70	1.89±0.72	1.90±0.73	0.497	1.80±0.80	1.81±0.69	1.85±0.71	1.91±0.70	1.92±0.74	<0.001
**B**												
** **	**Grade of Hepatic Steatosis**											
**Factors**	**No/Mild (n = 2736)**			**Moderate (n = 277)**			**Severe (n = 183)**			**p (trend)**		
hs-CRP, mg/L	0.16±0.23			0.19±0.21			0.25±0.26			<0.001		
WBC count, (10^3^/mm^3^)	5.99±1.57			6.28±1.55			6.74±1.70			<0.001		
Neutrophil count (10^3^/mm^3^)	3.42±1.19			3.57±1.17			3.92±1.27			<0.001		
Eosinophil count (10^3^/mm^3^)	0.16±0.15			0.16±0.14			0.19±0.15			0.001		
Basophil count (10^3^/mm^3^)	0.02±0.02			0.02±0.02			0.02±0.03			0.69		
Monocyte count (10^3^/mm^3^)	0.40±0.15			0.42±0.15			0.46±0.19			<0.001		
Lymphocyte count (10^3^/mm^3^)	1.96±0.60			2.04±0.59			2.11±0.63			<0.001		
NLR	1.87±0.83			1.84±0.69			1.99±0.81			0.001		

WBC: white blood cell; NLR: neutrophil lymphocyte ratio

**Fig 2:**
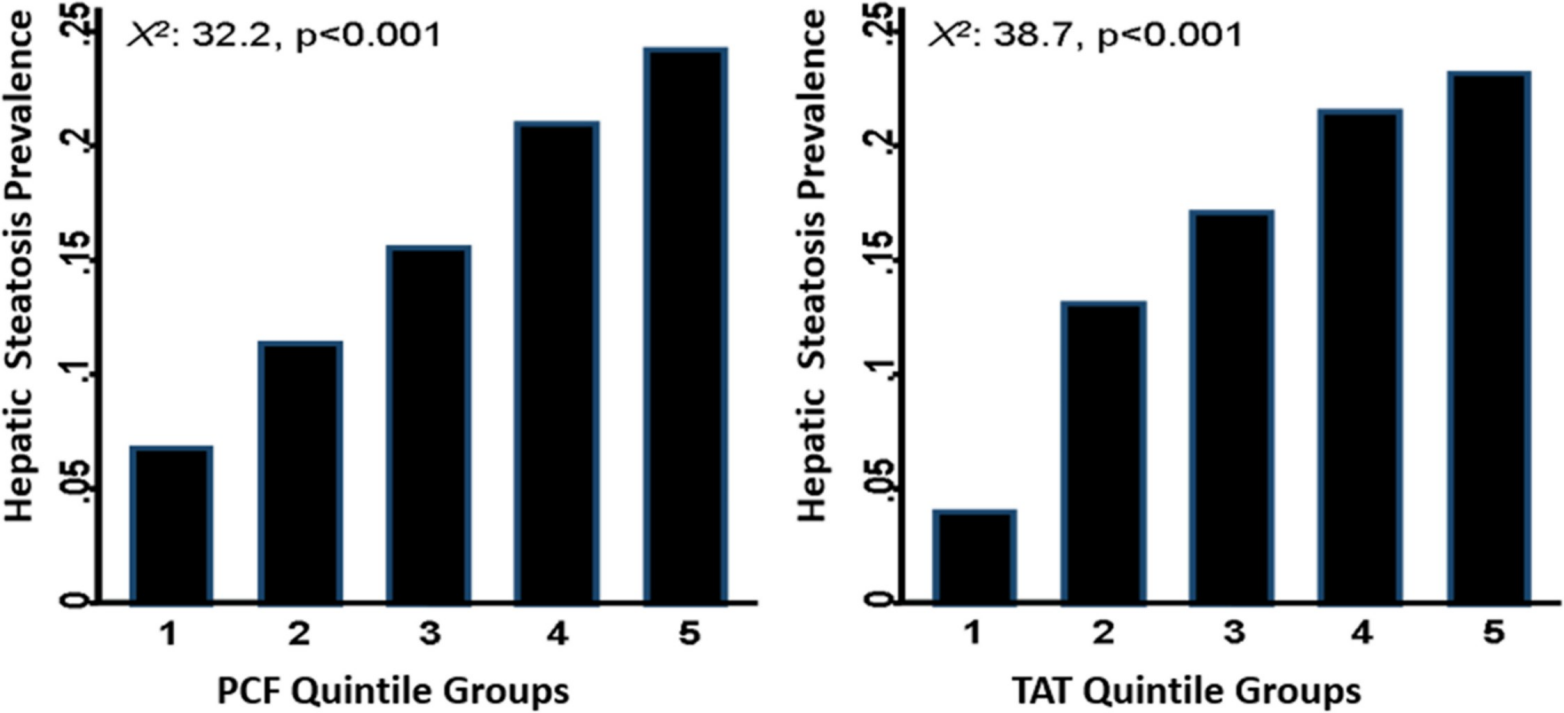
Prevalent hepatic steatosis across PCF, TAT volume quintiles. Subjects with higher quintile of PCF and TAT volume were more likely to have prevalent Hepatic Steatosis (both p<0.001 for *X*^2^ test: 32.2 & 38.7, respectively).

**Fig 3:**
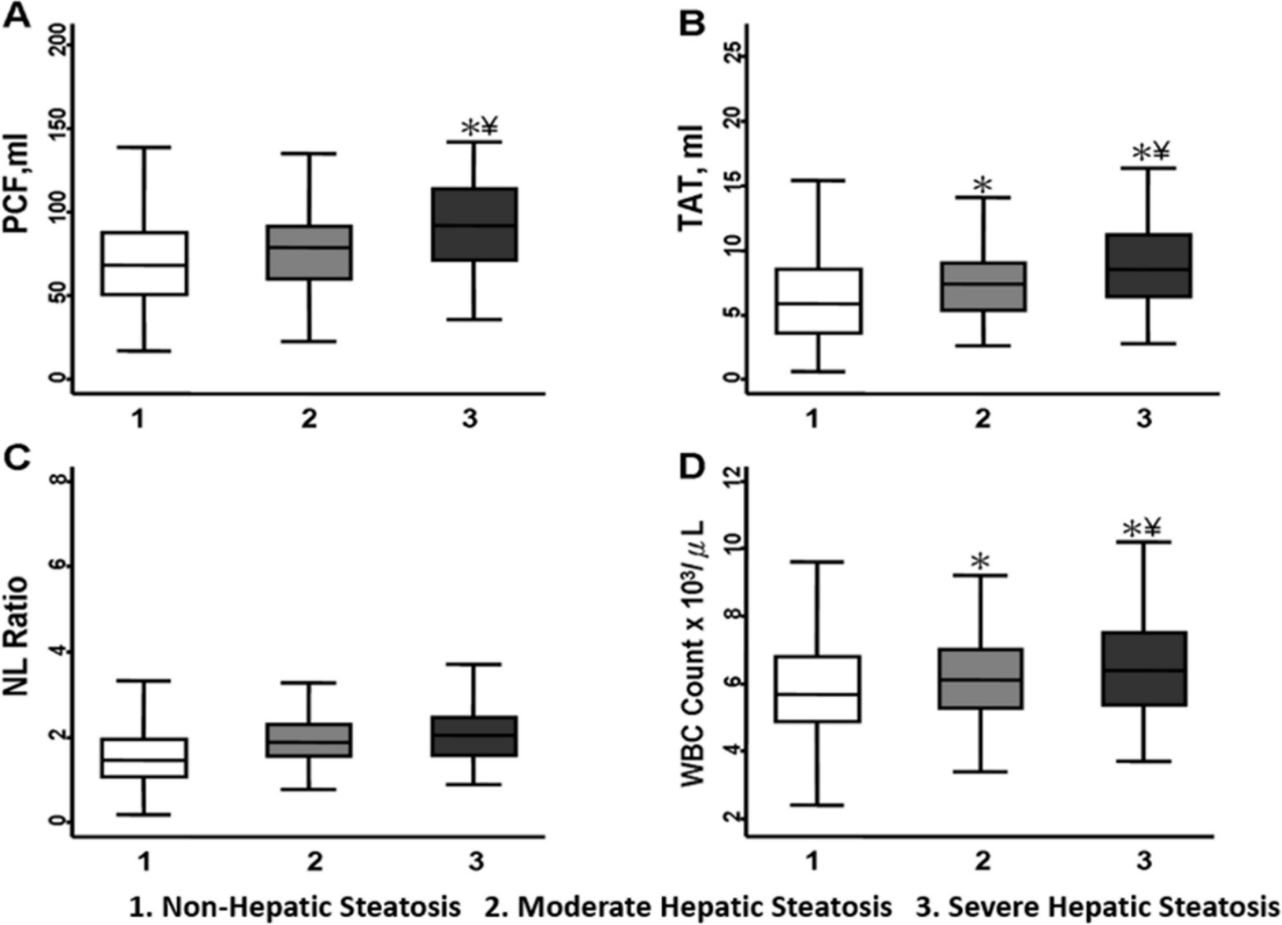
PCF, TAT, NLR, total leukocyte counts across hepatic steatosis category. Higher severity of Hepatic Steatosis [categorized as (1) No/Mild, (2) Moderate, and (3) Severe] was associated with greater burden of visceral adiposity (A and B) and higher total leukocyte counts (D), but was not associated with NLR (C). *p<0.05 compared to Non- Hepatic Steatosis group, ¥p<0.05 compared to Moderate Hepatic Steatosis group.

**Fig 4:**
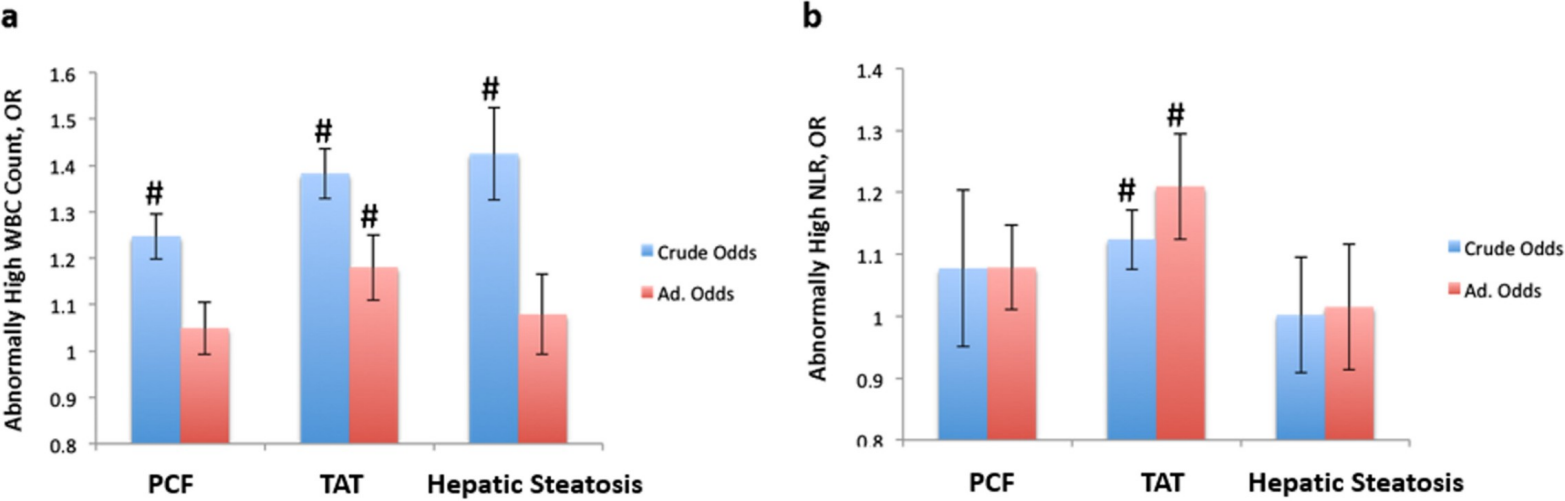
**Odds of abnormally high WBC (a) and NLR (b) in relation to increasing PCF, TAT, and presence of hepatic steatosis.** The crude and adjusted (adj.) odds ratios (OR) for PCF, TAT and Hepatic Steatosis in identifying abnormally high total leukocyte counts (WBC, cut-off: >6.9 * 10^3^/μL) and NLR (cut-off: >2.51).

**Fig 5:**
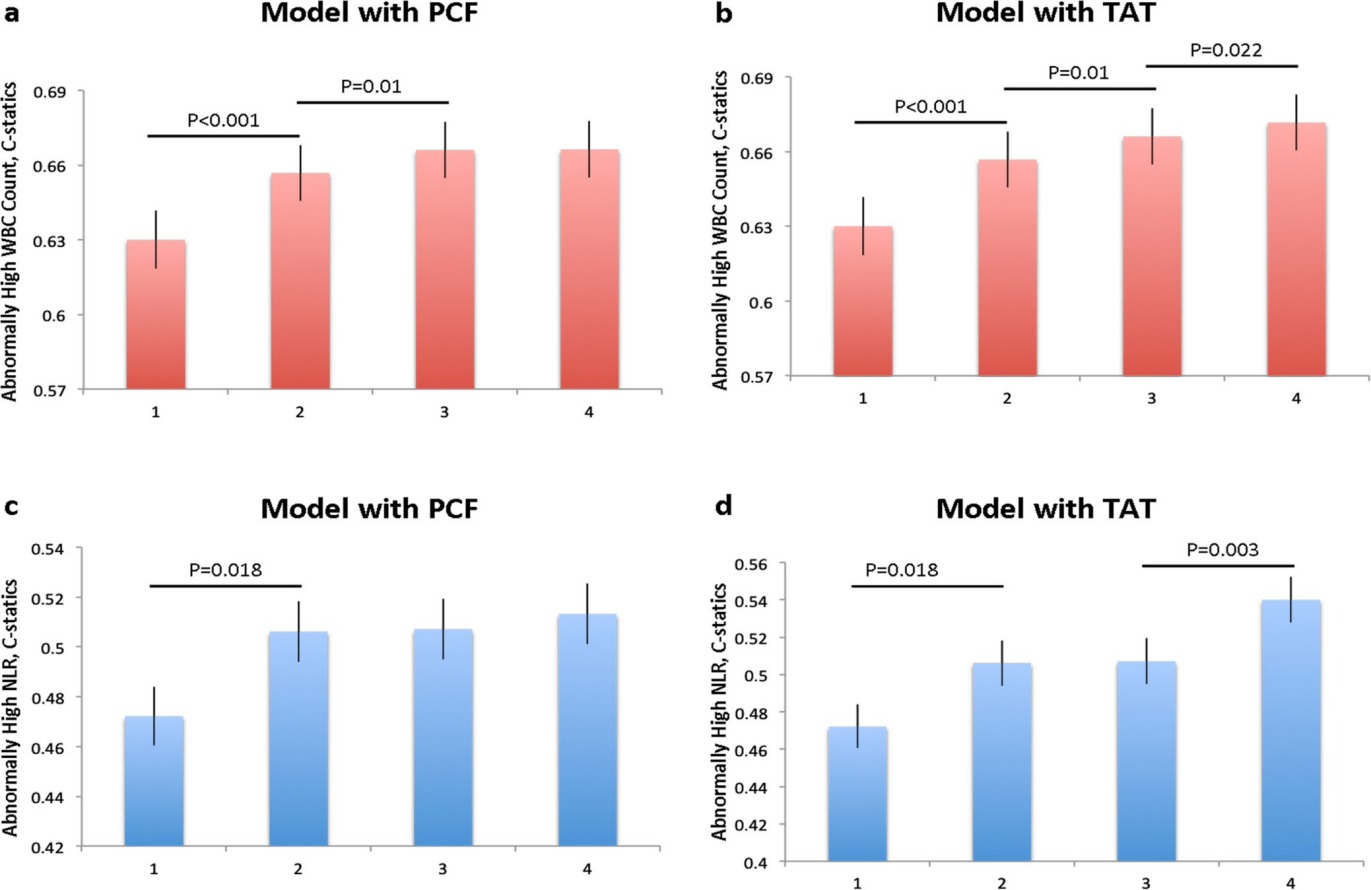
The prediction value of abnormally high WBC, NLR by PCF, TAT. The C-statistics of PCF, and TAT in the model prediction for abnormally high total leukocyte counts (WBC, cut-off: >6.9 * 10^3^/μL)^18^ and NLR (cut-off: >2.51). Again, TAT demonstrated significant incremental value beyond conventional cardiovascular risk factors by C-statistics from 0.66 to 0.67 for abnormal WBC, and 0,50 to 0.54 for abnormal NLR (c, d). #p<0.05 in fully adjusted models.

Volumes of PCF/TAT and hepatic steatosis severity were positively correlated with hs-CRP, total WBC counts, and higher levels of neutrophils, eosinophils, monocytes, and lymphocytes (Table 3). In addition, subjects with higher quintile of PCF and TAT volume were associated with higher prevalence of hepatic steatosis (Fig 2). Volumes of PCF/TAT correlated with hs-CRP (r = 0.18, 0.21 for PCF/TAT, both p<0.001). A graded increase of hs-CRP was also observed across hepatic steatosis categories (0.17, 0.19 & 0.25, trend p<0.001). Further, hs-CRP was positively associated with total WBC count (r = 0.24) and several individual white blood cell components (r = 0.27, 0.16, 0.24 for neutrophils, monocytes, and NLR, respectively, all p<0.001). Higher PCF and TAT along with substantially higher total WBC counts were observed across hepatic steatosis categories (Fig 3). In multi-variate models, adjustment for age, gender (Model 1), total body mass index (BMI, Model 2) and other clinical covariates attenuated the relationships between visceral adiposity (PCF/TAT), hepatic steatosis and hs-CRP, WBC or its differential blood cell types, especially for PCF and hepatic steatosis (Table 4). By defining abnormal total WBC count and NLR at a cut-off of 5,900 x 10^3^μL and 2.51, respectively, higher PCF and TAT burden were significantly associated with higher risk of abnormally high WBC count (Crude OR: 1.25 [95% CI: 1.1.15–1.35] & 1.38 [95% CI: 1.28–1.49] for PCF & TAT, both p<0.001) and showed modest associations with abnormally high NLR (Crude OR: 1.08 [95% CI: 0.98–1.18] & 1.12 [95% CI: 1.02–1.23], p = 0.126 & 0.014), respectively. In fully adjusted models, only higher TAT was independently associated with abnormally high total WBC count and NLR (adj. OR: 1.18 [95% CI: 1.05–1.33] & 1.21 [95% CI: 1.05–1.39], p = 0.006 & 0.007 for total WBC and NLR, respectively) (Fig 4A and 4B). Existence of moderate/severe hepatic steatosis (vs. no or mild) was also associated with abnormally high total WBC count (Crude OR: 1.42 [95% CI: 1.24.15–1.64, P<0.001]) but not abnormal NLR (Crude OR: 1.00 [95% CI: 0.83.15–1.20, P = 0.98); however, the association between hepatic steatosis and high WBC count was attenuated in fully adjusted models (adj. OR: 1.08, p = 0.34) (Fig 4A and 4B). Associations between TAT and total WBC, neutrophil, monocyte count, and NLR remained statistically significant in fully-adjusted models (Table 4, Model 3, all p<0.05). Fig 5A-5D demonstrate changes in the c-statistic for models of total WBC count and NLR as more co-variates were added: (1) age, gender, BMI; (2) model 1 + fasting sugar, LDL, HDL and eGFR; (3) model 2 + history of hypertension, diabetes, cardiovascular disease + lifestyle behaviors (active smoker and regular alcohol use); and (4) model 4 + PCF or TAT. In the final model 4, addition of TAT—but not PCF—statistically significantly increased the C-statistic for both total WBC count and NLR (Fig 5A-5D).

**Table 3:** The regression models for both visceral adipose tissue, hepatic steatosis (moderate-severe degree) with WBC, it`s subtypes and NLR.

**A**				
** **	**PCF, ml**		**TAT, ml**	
**Factors**	**ß-Coef**	**p value**	**ß-Coef**	**p value**
hs-CRP, mg/L	0.09	0.002	0.12	<0.001
WBC count (10^3^/mm^3^)	0.224	<0.001	0.32	<0.001
Neutrophil count (10^3^/mm^3^)	0.127	<0.001	0.197	<0.001
Eosinophil count (10^3^/mm^3^)	0.01	<0.001	0.015	<0.001
Basophil count (10^3^/mm^3^)	0	0.941	0	0.966
Monocyte count (10^3^/mm^3^)	0.015	<0.001	0.024	<0.001
Lymphocyte count (10^3^/mm^3^)	0.059	<0.001	0.078	<0.001
NLR	0.019	0.164	0.042	0.002
**B**				
** **	**Hepatic Steatosis (Moderate-Severe Degree)**			
**Factors**	**Coef**		**p value**	
hs-CRP, mg/L	0.07		0.003	
WBC count (10^3^/mm^3^)	0.35		<0.001	
Neutrophil count (10^3^/mm^3^)	0.23		<0.001	
Eosinophil count (10^3^/mm^3^)	0.014		0.007	
Basophil count (10^3^/mm^3^)	─		─	
Monocyte count (10^3^/mm^3^)	0.02		<0.001	
Lymphocyte count (10^3^/mm^3^)	0.077		<0.001	
NLR	0.03		0.01	

WBC: white blood cell; NLR: neutrophil lymphocyte ratio

**Table 4:** Multivariate adjustment models in the association between both visceral adipose tissue, moderate-to-severe hepatic steatosis, WBC, it`s subtypes and NLR.

**Multi-variate Model**	**Multi-variate Model 1**				**Multi-variate Model 2**				**Multi-variate Model 3**			
**Factor**	**PCF, ml**		**TAT, ml**		**PCF,ml**		**TAT, ml**		**PCF, ml**		**TAT, ml**	
	**ß-Coef**	**p value**	**ß-Coef**	**p value**	**ß-Coef**	**p value**	**ß-Coef**	**p value**	**ß-Coef**	**p value**	**ß-Coef**	p value
hs-CRP, mg/L	0.06	0.032	0.1	0.001	0.06	0.23	0.06	0.094	0.05	0.52	0.08	0.38
WBC count (10^3^/mm^3^)	0.243	<0.001	0.339	<0.001	0.107	<0.001	0.208	<0.001	0.135	0.012	0.155	0.011
Neutrophil count (10^3^/mm^3^)	0.15	<0.001	0.242	<0.001	0.067	0.004	0.169	<0.001	0.065	0.143	0.106	0.04
Eosinophil count (10^3^/mm^3^)	0.008	0.005	0.01	0.002	0.006	0.099	0.007	0.056	0.004	0.42	0.157	0.073
Basophil count (10^3^/mm^3^)	0	0.774	0	0.537	0	0.945	0	0.635	0	0.718	0.001	0.27
Monocyte count (10^3^/mm^3^)	0.015	<0.001	0.021	<0.001	0.007	0.026	0.013	<0.001	0.007	0.243	0.017	0.022
Lymphocyte count (10^3^/mm^3^)	0.059	<0.001	0.068	<0.001	0.023	0.054	0.023	0.089	0.033	0.12	0.028	0.248
NLR	0.028	0.052	0.072	<0.001	0.02	0.218	0.081	<0.001	0.011	0.52	0.062	0.002
**Multi-variate Model**	**(Moderate-to-Severe) Hepatic Steatosis**											
**Factor**	**Multi-variate Model 1**				**Multi-variate Model 2**				**Multi-variate Model 3**			
	**Coef**		**p value**		**Coef**		**p value**		**Coef**		**p value**	
hs-CRP, mg/L	0.07		0.004		0.01		0.56		0.004		0.71	
WBC count (10^3^/mm^3^)	0.28		<0.001		0.13		0.01		─		─	
Neutrophil count (10^3^/mm^3^)	0.19		<0.001		0.1		0.012		─		─	
Eosinophil count (10^3^/mm^3^)	─		─		─		─		─		─	
Basophil count (10^3^/mm^3^)	─		─		─		─		─		─	
Monocyte count (10^3^/mm^3^)	0.02		0.001		─		─		─		─	
Lymphocyte count (10^3^/mm^3^)	0.06		0.007		─		─		─		─	
NLR	0.04		0.014		0.03		0.032		─		─	

Model 1: adjusted for age, gender; Model 2: adjusted for age, gender, BMI; Model 3: adjusted for age, gender, BMI, multi-variate models (MV); BMI: body mass index. MV: systolic blood pressure, pulse rate, fasting glucose, HDL, LDL, eGFR, medical history of diabetes, hypertension, cardiovascular disease, current smoker, alcohol use.

## Discussion

In this large cohort of subjects who underwent a cardiovascular health survey in Taiwan, we demonstrate that 3 measures of visceral adiposity—PCF, TAT and hepatic steatosis—are associated with circulating WBC counts and the systemic inflammatory marker hs-CRP. Moreover, compared to PCF, TAT appears to be more strongly related to WBC counts and the NLR, an integrative measure of generalized inflammation, even after accounting for clinical co-variates. Further, we observed that TAT added independent and incremental value in identifying abnormally higher total WBC counts and NLR. Our study suggests that regional ectopic fat deposition of the thoracic aorta (TAT) is associated with systemic inflammation and altered leukocyte numbers above and beyond clinical characteristics and body anthropometrics.

Peripheral total leukocyte counts (WBC) and its subtypes have previously been associated with cardiometabolic risk factors including dyslipidemia, obesity, and metabolic syndrome [20–22]. Higher total WBC counts have been further associated with clinical events such as incident type 2 diabetes and increased mortality after acute coronary syndromes [23, 24]. These and other relevant studies illustrate how atherosclerotic cardiovascular disease is a complex multi-factorial process in which inflammation plays a major role. Abnormal adipose tissue distribution and function are believed to contribute to atherosclerosis, an effect which may be mediated by inflammation. At the adipose tissue level, inflammation is complex and characterized by dynamic populations of infiltrating neutrophils, macrophages, mast cells, and lymphocytes [7, 25]. Among these, adipose tissue macrophages (ATMs), may be the most important drivers of obesity-related chronic inflammation [26]. In both mouse models and human subjects, adipocyte hypertrophy induced by excessive caloric intake triggers macrophage accumulation in visceral adipose depots where they form so-called “crown-like structures” (CLSs) around dead adipocytes [27, 28]. These infiltrating macrophages express the pro-inflammatory M1 phenotype and secrete a variety of inflammatory cytokines such as tumor necrosis factor-α, interleukin-6 and monocyte chemoattractant protein-1, leading to up-regulated circulating leukocytes and trigger systemic inflammatory responses [29]. Importantly, these pro-inflammatory cytokines may act locally on adipocyte function and systemically on other tissues to promote the pathogenesis of cardiometabolic disease [30]. In our current work, we demonstrate that both increased PCF and TAT (but not hepatic steatosis) were independently associated with higher total leukocyte counts after accounting for clinical co-variates. Of note, only TAT showed independent and incremental value in predicting abnormally high total leukocyte counts (5900 x 10^3^μL) and higher NLR (>2.51).

Recently, the NLR has been proposed as a simple, clinically available and inexpensive marker of systemic inflammation, which is helpful for risk stratification of patients with cardiovascular diseases, diabetes mellitus, metabolic syndrome [11, 31], and for subsequent adverse cardiovascular events following myocardial infarction [32]. Neutrophils may contribute to acute myocardial injury by degranulation and secretion of inflammatory cytokines and proteases, resulting in tissue damage and reperfusion injury [33, 34]. Furthermore, neutrophils can also invade atherosclerotic plaques and trigger oxidative stress by releasing superoxide radicals, proteolytic enzymes & arachidonic acid derivatives, which all make atherosclerotic plaques more vulnerable [35]. On the other hand, lymphocytes play an important role in regulating the inflammatory response and are suppressed by corticosteroids in response to the stress of acute coronary syndrome [36]. In previous studies, however, increased BMI was associated with total WBC and individual sub-types but not with NLR [37–39]. A multi-center study revealed that NLR is positively correlated with hepatic steatosis activity score, pro-inflammatory cytokines and CRP [40], though in our multi-variate analysis the associations between hepatic steatosis and all types of leucocytes were attenuated, indicating that hepatic steatosis might play a lesser role in mediating systemic leucocytes circulation. In aggregate, our work further expands on these previous observations to show that TAT—a measure of regional visceral adiposity—, instead of PCF or hepatic steatosis, is tightly associated with NLR beyond clinical covariates, BMI and lipid profiles. These findings are also consistent with ectopic visceral fat contributing to elevated cardiovascular risk in “metabolically obese” normal-weight individuals [41, 42]. While both TAT and PCF remained independently related to total leucocyte counts and NLR, only TAT statistically significantly improved the c-statistic of our fully-adjusted model. This finding may be partly explained by anatomical difference. Elicited abdominal visceral adipose tissue inflammation close to portal circulation may release fatty acids and cytokines that directly affect liver (the end-organ), which is considered as the key factor of hepatic steatosis and insulin resistance [43]. Instead, PCF is anatomically confined within the pericardial sac, which was believed to be the source of pro-inflammatory adipokines near coronary arteries and myocardium. Recent studies have demonstrated close relationships between PCF and pathogenesis of atherosclerosis or certain degree of myocardial dysfunction [44, 45] Compared to PCF and hepatic steatosis, TAT may play a more important role in systemic inflammatory process, partly due to the fact that cytokines and pro-inflammatory mediators from TAT can more easily diffuse and distribute through the adventitia layer across the arterial wall to enter the systemic circulation [12, 46]. This data together with our previous findings underscores the possibility of using TAT as important metabolic surrogate marker which may provide additional information in identifying subjects at higher risk of cardiovascular disorders. Further investigation is warranted to explain why different locations of ectopic visceral fat may be differentially associated with systemic inflammation.

This study has some limitations. Because it is a cross-sectional study, causal inferences cannot be made and the possibility of residual or unknown confounding cannot be excluded. Additionally, our subjects may not represent the general population in Taiwan, since they were participating in a voluntary health survey and were not randomly selected. Our study was disproportionally male, and so further study in larger female populations is warranted. Finally, our study represented only a single ethnicity (Taiwanese). In light of racial and ethnic differences in NLR [47], future studies should examine these relationships in other populations.

## Conclusion

Our study results indicate that greater visceral fat depots appear to be associated with higher WBC counts and individual cell types independent of body mass index and other traditional risk factors; however, compared to ectopic fat within the pericardial sac, visceral fat surrounding the aorta is more strongly associated with the NLR.

## Supporting information

S1 Fig The relationship between total leukocyte, neutrophil, monocyte, NLR and groups according to low or high BMI (<24.3, ≥24.3kg/m2) and low and high PCF/TAT categories (<71, ≥71ml for PCF; <6.4, ≥6.4ml for TAT) based on median values. Total WBC and the proportion of neutrophil and monocyte tended to increase across BMI and both PCF and TAT groups (all p for trend: <0.05) For NLR, the association across BMI/TAT categories was statistically significant but was not for BMI/PCF categories (p = 0.008 vs. p = 0.196 for TAT vs. PCF, respectively).
(PPTX)

## Abbreviations

PCF: Pericardial adipose tissue
TAT: thoracic peri-aortic adipose tissue
VAT: visceral adipose tissue
MDCT: multi-detector computed tomography
hs-CRP: high-sensitive C-reactive protein
BMI: body mass index
BSA: body surface area
DBP: diastolic blood pressure
SBP: systolic blood pressure
eGFR: estimated glomerular filtration rate
HbA1c: glycosylated hemoglobin level
HDL: high-density lipoprotein
LDL: low-density lipoprotein
