# Supplementary figures and images for: Associations between CT-determined visceral fat burden, hepatic steatosis, circulating white blood cell counts and neutrophil-to-lymphocyte ratio
Source: PLoS One. 2018 Nov 20;13(11):e0207284. doi: 10.1371/journal.pone.0207284 (PMC6245737; doi:10.1371/journal.pone.0207284)

## Slide 1
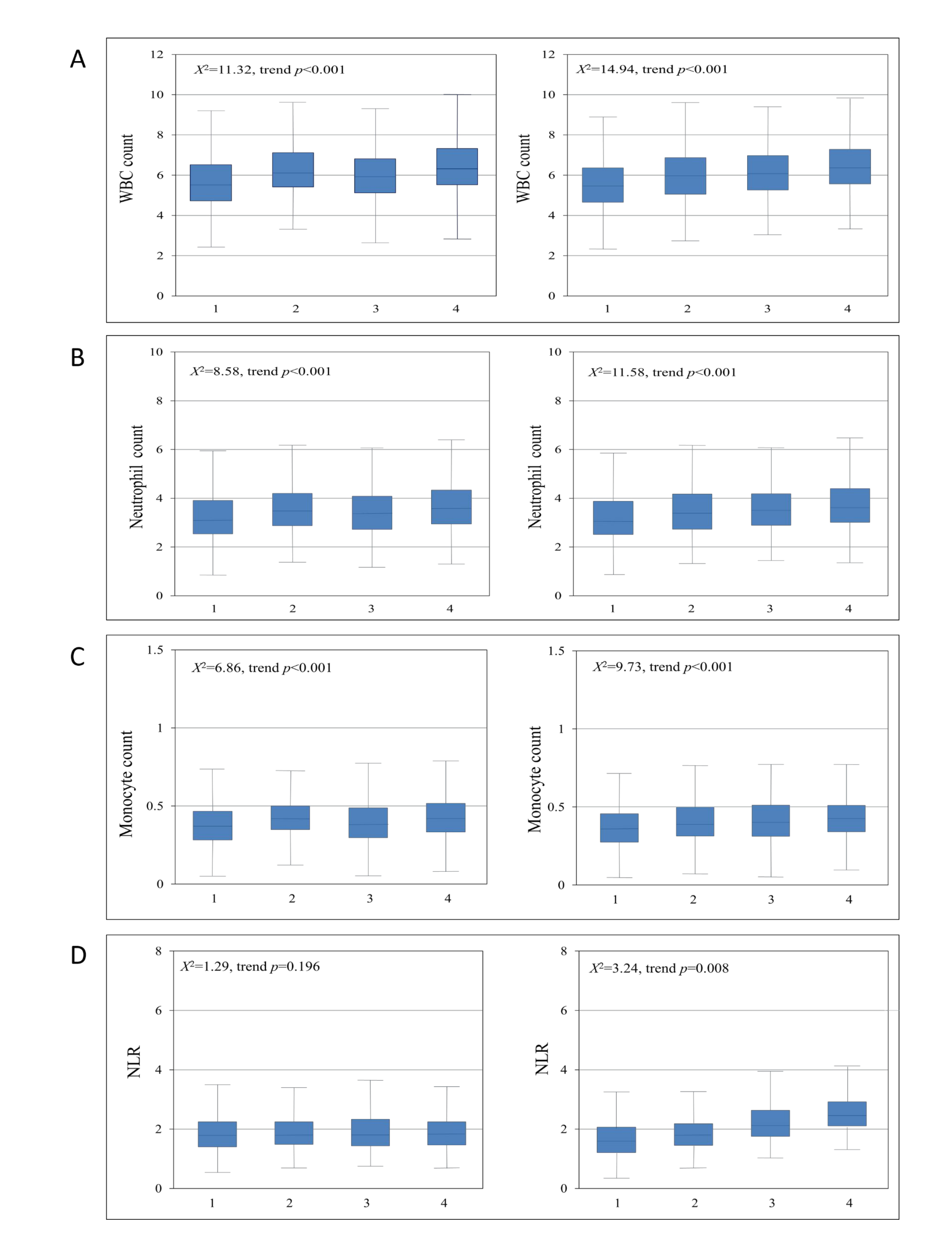

Supplement: S1 Fig — Total WBC and the proportion of neutrophil and monocyte tended to increase across BMI and both PCF and TAT groups (all p for trend: <0.05) For NLR, the association across BMI/TAT categories was statistically significant but was not for BMI/PCF categories (p = 0.008 vs. p = 0.196 for TAT vs. PCF, respectively). (PPTX) [file pone.0207284.s001.pptx]
